# Comparing the racialization of Central-East European migrants in Japan and the UK

**DOI:** 10.1186/s40878-021-00239-z

**Published:** 2021-07-14

**Authors:** Špela Drnovšek Zorko, Miloš Debnár

**Affiliations:** 1 University of Warwick, Coventry, UK; 2 Ryukoku University, Kyoto, Japan

**Keywords:** Whiteness, Central-East European migration, Racialization, UK, Japan

## Abstract

The article deploys the lens of the race-migration nexus (Erel et al., Ethnic and Racial Studies 39:1339–1360, 2016) to compare the racialization of migrants in the UK and Japan. It draws on qualitative data on the experiences of Central-East European (CEE) migrants in the two countries to unpack how whiteness is constructed in relation to different histories and patterns of immigration in each national context. While CEE migrants in Japan benefit from being perceived as implicitly white and Western ‘foreigners’, their whiteness represents a form of enduring exclusion from the ethno-nationalist Japanese society. In the UK, changing political contexts and internal European hierarchies of whiteness contribute to CEE migrants’ ambiguous position in an increasingly anti-migrant society. By comparing the mechanisms of racialization in each country through the analytics of visibility and exclusion, the article furthers ongoing debates about the intersections of race and migration. It furthermore extends the comparative analysis of whiteness to a non-Western setting, making a significant contribution to the study of local/global articulations of race.

## Introduction: race and migration in Japan and the UK

This article compares the ways in which local/global dynamics of race and disparate histories of immigration produce distinct processes of racialization in Japan and the UK by analyzing the experiences of migrants from the Central-East European (CEE)^1^ region in both countries. It contributes to the special issue’s focus on diversity and complexity by comparing subjective experiences of racialization, arguing for a more nuanced understanding of how migrants experience visibility and exclusion in distinct national contexts. The article further contributes to the latest developments in research on race, including the sub-field of whiteness studies, by analyzing constructions of whiteness in two cases that go beyond the better-studied examples of white-majority populations in the West on the one hand and the postcolonial dynamics of migration on the other.

The two case studies, conducted in Japan (Miloš Debnár) and the UK (Špela Drnovšek Zorko), offer fertile ground for comparing how racialization is linked to migration. In Japan, an “ethno-nationalist immigrant society” (Liu-Farrer, 2020) currently experiencing increasing rates of immigration, inclusion remains implicitly racialized. Japanese national identity is constructed in a way that conflates race, culture, citizenship and language, a conceptualization that continues to be reproduced rather than seriously contested (Weiner, 1997). In the UK, a white-majority society, definitions of Britishness were forced to expand with the post-war arrival of labor migrants from the rapidly declining Empire who joined existing black and Asian populations in the British Isles (Fryer, 1984; Hirota et al., 2019). With the rise of nativist politics reflected in the Brexit vote, however, migrants and racialized “internal others” (Virdee & McGeever, 2018, p. 2) are being increasingly excluded from the British ideal.

To account for specificities in how notions of race intersect with migration in each country, we draw on the concept of the “race-migration nexus” (Erel et al., 2016), which highlights the ways in which racialized subjectivities are constituted through immigration regimes and discourses. By comparing the experiences of CEE migrants using the analytical categories of visibility and exclusion, which emerged as salient themes in our data, the article contributes to our understanding of how “the relations between race and migration are currently configured” (Erel et al., 2016, p. 1341) in two highly varied contexts, as well as which differences make a difference (introduction to this special issue) in the UK and Japan. To date, most research on contemporary CEE migrants has focused on Western Europe, including the UK, where internal European hierarchies and claims to implicitly white Europeanness have often positioned migrants from the CEE region “inbetween” (Botterill & Burrell, 2019, p. 24) the less spurious whiteness of Western or Northern Europeans and the racial Otherness of non-Europeans. Comparison with the Japanese context provides the opportunity to explore constructions of whiteness in a setting where racial hierarchies are aligned differently than in Western Europe. Our analysis reveals that migrants’ lived experiences of racialization vary as a result of differences in how whiteness is assembled and the connotations it carries in the two countries, as well as how it is positioned in relation to the dominant social group. Nevertheless, in both cases migrants experience a form of differentiation linked to practices and prevailing discourses about immigration, which contribute to “co-constituting hierarchies an [*sic*] ‘us’ and ‘them’ in essentialized terms” (Erel et al., 2016, p. 1343).

To explore what the experiences of CEE migrants in UK and Japan can tell us about constructions of race in relation to migration, we first present the theoretical background to the racialization of CEE migrants and constructions of whiteness, and develop our argument for why the race-migration nexus presents a useful lens for comparing such different contexts. We then provide further background on the two case studies and set out our methodology, before moving on to compare empirical data in two analytical sections exploring migrants’ racialized visibility and exclusion.

## Contextual constructions of whiteness and the race-migration nexus

The question of how migration contributes to racialization has emerged as a key concern for sociologists and geographers interested in construction of race (De Genova, 2016; Erel et al., 2016; de Noronha, 2019). The main contention of such approaches is that the process of racialization – “how the construction of race is shaped historically and how the usage of that idea forms a basis for exclusionary practices through cultural or political processes” (Erel et al., 2016, p. 1342) – is not only contextual and historically specific, but also shaped in and through discourses and practices related to migration. Rather than being determined solely by either sending or receiving contexts, migrants’ experiences of racialized difference are therefore a “transnational co-production” (Nowicka, 2018) embedded in both local and global dynamics and articulations of race (Loftsdóttir, 2017, p. 71).

### The racialization of Central-East European migrants

The experiences of postsocialist CEE migrants in Western Europe, and the extent to which these experiences interrogate long-held assumptions about the global structural advantages of whiteness, have proven a productive arena for grappling with the contradictions of racialization in relation to migration (Botterill & Burrell, 2019; Favell, 2008; Krivonos, 2020; Loftsdóttir, 2017; McDowell, 2009; Ryan, 2010). Despite the initial promise of EU expansion in 2004 and 2007 leading to “the end of the East/West division of the continent” (Favell, 2008, p. 264) and a new era of mobility for CEE EU citizens endowed with the same rights as their Western European counterparts, the reality has proven more complex, and “cultural distinctions and (even) racialised subordinations have been commonplace among the experiences of CEE movers” (Favell, 2008, p. 264). The parenthetical “even” points to the extent to which CEE migrants have been viewed as a case study for examining “the various (in) visibilities and performativities of whiteness” (Botterill & Burrell, 2019, p. 24) as a constructed, relational, and permeable category rather than an intrinsic characteristic of certain groups (McDowell, 2009, pp. 28–29). As Fox, Moroşanu and Szilassy sum up their research on the racialization of post-2004 migrants in the UK, “nominally shared whiteness between migrant and majority has not exempted these current cohorts of migrants from the sorts of racialization found in other migrations” (Fox et al., 2012, p. 682), a reference to the long-standing association between post-war migration to Western Europe and racial Otherness.

Notions of Europeanness play a significant role in the position of CEE migrants, with Europeanness still predominantly racialized as white (El-Tayeb, 2011). Hierarchies of Europeanness also function along an East/West, as well as North-South (Antonucci & Varriale, 2020), divide. Scholars have explored how long-established categorizations positioning Eastern Europe as endlessly ‘catching up’ with the West (Dzenovska, 2018) shape contemporary CEE migrants’ opportunities in Western Europe, as well as how post-Cold War inequalities both within and outside the EU contribute to entrenching historical divisions between the East and West of the continent. In Helsinki, for instance, Russian-speaking migrants’ experiences are shaped both by post-Soviet economic inequalities and Finland’s alignment with Western Europe in opposition to Russia (Krivonos, 2020). In Iceland, new patterns of immigration and media narratives about criminality shape the perceptions of CEE migrants within racialized schemas, leading to questions about Polish migrants’ “race” (Loftsdóttir, 2017, p. 73). In post-Brexit Britain, meanwhile, “orientalist hierarchies” have placed Poles, who represent the largest CEE community in the UK, in a liminal position: “more ‘western’ than Commonwealth or Middle Eastern populations, but not as western as other, nearer, Europeans” (Botterill & Burrell, 2019, p. 25). At the same time, such experiences of “ambiguous whiteness” (Lapiņa & Vertelyte, 2020) do not exempt CEE migrants from reproducing racialized hierarchies and exclusions in the pursuit of attaining full European whiteness (Drnovšek Zorko, 2019; Krivonos, 2020; Tudor, 2017).

### CEE migrants in the UK: changing contexts of racialization

In the UK, the post-2004 ‘new Europeans’ were initially welcomed by policy-makers as a pool of migrant labor needed to fill specific industry shortages (Fox et al., 2012). Scholars have pointed to the continuities between such ‘managed migration’ and the policies that historically favored immigration from the Baltics over the Commonwealth (McDowell, 2009). For some migrants from the region, especially earlier arrivals, this was borne out by employers who claimed to favor CEE migrants because they were “white” and “hardworking” (Parutis, 2011). However, as nativist attitudes and anti-migrant hostilities increasingly became part of the British political mainstream, particularly after the 2016 Brexit vote which was “principally framed … by issues of immigration, race and difference” (Valluvan, 2019, pp. 1–2), CEE migrants have become increasingly visible in the public sphere (Botterill & Burrell, 2019). Fox, Moroşanu and Szilassy thus contrast the initial racialized *inclusion* of CEE migrants in policy with their racialized *exclusion* in the tabloid media (and political discourse) as a form of racism based not on physical characteristics but on presumed cultural differences (Fox et al., 2012, pp. 685–691).

CEE migrants’ ambivalent whiteness therefore is a complex amalgamation of visible and audible markers of difference, including names, accents, dress, and at times, a “foreign look” (Botterill & Burrell, 2019; Rzepnikowska, 2019, p. 70). Rzepnikowska (2018) contrasts the experiences of Polish migrant women in Barcelona, where Poles are viewed as culturally proximate “model minorities”, with the UK, where media discourses about Polish and other CEE migrants have frequently portrayed them as a strain on social services (Rzepnikowska, 2018, p. 852). Yet the experiences of ‘new’ EU migrants represent only part of the picture of racialized exclusion and cannot be decoupled from the long history of racism experienced by racial minorities and illegalized migrants. Since 2012, the UK government’s strategy of creating a ‘hostile environment’ for irregular immigration has increasingly scapegoated all non-citizens, re-enforcing existing racialized exclusions while targeting new populations for immigration control (Goodfellow, 2019). The rise in hate crimes experienced by both CEE and long-settled minority communities around the time of the Brexit referendum further underscored the saliency of both old and more novel forms of exclusion.

The case of CEE migrants in Western European countries allows us to explore constructions of whiteness in contexts where whiteness is a hegemonic national identity, and where it is largely aligned with global racial hierarchies of white superiority. There is, however, a distinct lack of research on how notions of whiteness are configured in contexts where such global racial hierarchies are implicitly present but where whiteness is not necessarily hegemonic and where Western racial hierarchies are contested. By adding the case of Japan to the discussion of the racialization of CEE migrants, we can expand the analysis of local/global articulations of whiteness to a non-Western yet highly developed country, with its own historical constructions of race and identity.

### Whiteness and migration in the context of Japan

The case of Japan demonstrates how whiteness was both emulated as a norm of progress, modernization, or beauty, and at the same time contested as a governing principle of the global hierarchy (Bonnett, 2000). Bonnett argued that there is a “complex and tense” relationship between assimilating and refusing whiteness (Bonnett, 2000, p. 75), resulting in ambiguity, where “the white must be simultaneously ‘like us’ and ‘not like us’, both foreign and not foreign” (Bonnett, 2000, p. 71). On the one hand, Japan was the first non-Western adopter of the Western notion of race (Kowner, 2018) and the notions of race and racial hierarchy were strategically assimilated in Japan as a way to distinguish them both from other Asians and Westerners (Kawai, 2015). Japan aimed to become a country that can be seen both as “aligned to ‘the white club of nations’” and being perceived as different (Bonnett, 2000, p. 67). At the same time, however, there is a long history of refusing whiteness as a racialized identity of power signifying hierarchical subordination of all other racial groups, ranging from Japan’s early twentieth century efforts to challenge Western notions of race on the international level to post-war discourse of Japaneseness (Kawai, 2015; Kowner, 2018). Thus, while whiteness continues to represent a beauty standard and a norm to be emulated, which empowers its bearers, particularly in comparison to ‘others’ racialized as non-white and non-Japanese (Russell, 2017, p. 27), white “European-heritage people” have been also “partially dethrone [ed]” as “representatives of a superior ‘white race’” (Bonnett, 2000, p. 74) and the contemporary discourse on Japaneseness is an expression of widespread beliefs perceiving Japan, at least partly, as superior (Kowner, 2018, p. 101).

Recent studies on Europeans in Japan by Hof (2018, 2020) and Debnár (2016) highlight this complex and contradictory character of whiteness in the Japanese context by analyzing the experience of white European migrants. Debnár has argued that their whiteness works as “a double-edged sword” that both privileges them in certain occupational and cultural milieus while restricting their access to others (Debnár, 2016, pp. 160–170). Hof introduced the concept of “passive whiteness” to emphasize that whiteness often “merely functioned as a token, a trophy” (Hof, 2020, p. 11), and questioned the role of whiteness as a form of capital that white migrants can actively convert to other forms of capital benefiting their economic, social or cultural integration (see also Debnár, 2016; Miladinović, 2020).

Contemporary European migration to Japan is part of what continues to be a hesitant and still relatively limited engagement of Japan with accepting ‘foreign labor’ – a euphemism for ‘(im)migration’, which still represents a word stubbornly avoided and denied by political elites (Roberts, 2018). While the number of registered foreign nationals in Japan has almost tripled in the last three decades, the approximately 3 million foreign nationals residing in Japan in 2019 represented still only about 3% of the total population. This relative lack of post-war migration was one of the factors that co-constructed perceptions of Japan as an ethno-racially homogenous nation (Oguma, 2002), and such self-perceptions are seen as one of the major factors impeding further and more open acceptance of immigration as well as the integration of migrants. At the same time, however, it needs to be acknowledged that Japan has been becoming “an immigrant country” by increasingly providing “foreign nationals multiple legal channels to enter and legal paths and institutional frameworks to permanent settlement” in recent decades (Liu-Farrer, 2020, p. 8).

The issue of how CEE migrants are perceived in comparison to the UK raises different questions due to the often-contradictory status of whiteness in Japan, which continues to represent global hegemony yet is also being continuously contested, and historical associations between Westernness and whiteness. Second, while the presence of migrant communities has played a relatively limited role in the construction of race in the case of (post-war) Japan, it has played a significant role in the case of the UK. The article thus compares two contexts where local/global notions of race and whiteness are aligned differently and where migration has played different roles in such alignments, which has divergent consequences for the racialization of migrants.

### The race-migration nexus: linking immigration to constructions of race

To set the terms for such a comparison and elucidate how local/global articulations of race intersect with immigration discourses, we draw on the concept of the “race-migration nexus” developed by Erel et al. (2016). The race-migration nexus presents an analytical lens for investigating the diverse ways in which racialized subjectivities are co-constituted through immigration regimes and discourses. Rather than being mutually exclusive frameworks, the “nexi” identified by Erel et al. foreground the co-existence of different forms of racialization and act as “lenses on a camera in bringing particular constellations of the migration–racialization nexus into analytical focus”, underpinned by an attention to historical variability in how race is understood and enacted in specific contexts (Erel et al., 2016, p. 1342).

This approach allows us to engage with the specific forms of racialization unique to each case study while still comparing aspects of their underlying dynamics. In particular, the “complex migrations – differential racialization” nexus, which makes “visible the ways immigrants and settled communities emerge as uniquely racialized subjects through distinct, yet overlapping, hierarchies of legal status, gender, culture, class and social space” (Erel et al., 2016, p. 1347), provides a starting point for comparing the themes of visibility and exclusion that emerged as key issues in both case studies, albeit in different ways. Combined with an awareness of how distinct national discourses portray (im)migrants and “internal others” (Virdee & McGeever, 2018, p. 2) depending on their respective histories and patterns of immigration, this approach underscores the complexity of individual migrants’ experiences as shaped by diverse social locations and, at times, seemingly contradictory subject positions. The concept of the race-migration nexus thus allows us to compare the underlying mechanisms of racialization without equating either the position of CEE migrants in Japan and the UK or the immigration regimes and discourses that shape their experiences.

## Case studies and methodology

### Japan

In Japan, Central-East Europeans still represent a small population of so-called ‘newcomer’ migrants to Japan. With slightly less than 20,000 residents in 2019, they represented approximately one-fourth of all Europeans living in Japan (79,000 in 2019) and a mere fraction of 2.9 million foreign residents in total. However, the number of residents from CEE countries in Japan has increased approximately 19 times since the end of the 1980s, and they represent a relatively fast-growing minority. While there is very little research on the particular drivers of CEE migration to Japan, available statistics suggest that women entertainers or marriage migrants from Russia or Romania, student migration and subsequent long-term settlement, professional migration, and trailing migrants represent some of the patterns (Debnár, 2016, pp. 42–56).

It is important to note that in the case of Japan, there are no such substantial differences in the mobility regimes between certain groups of CEE countries as in the case of the UK and free intra-EU mobility.^2^ Thus, for the purposes of this paper, we include both EU and non-EU CEE nationals in the analysis of the Japanese case.

### UK

While migrants from CEE countries settled in the UK even before the 2004 EU expansion (McDowell, 2009), the UK government’s decision not to impose transitional arrangements for the free movement of ‘new Europeans’ triggered a new era of labor migration from the region. Immigration from the A8 countries (Czech Republic, Estonia, Hungary, Latvia, Lithuania, Poland, Slovakia, Slovenia), which joined the EU in 2004, rose from an estimated 167,000 in 2004 to 1,323,000 in 2018, while immigration from A2 countries (Bulgaria, Romania), which joined in 2007, rose from 42,000 in 2007 to 495,000 in 2018 – together accounting for more than half of all EU citizens in the UK (Vargas-Silva & Fernández-Reino, 2019).

Since the right to free movement allows EU nationals to live and work anywhere in the European Union (which included the UK at the time of the research), CEE migrants from EU countries have not been subject to the same immigration restrictions as migrants from non-EU countries. In order to account for this difference, the article only includes EU CEE nationals in the analysis of the UK case.

### Methodology

The paper utilizes data from two separate qualitative studies conducted in Japan and the UK (Table 1).

**Table 1:** Sample characteristics for the Japan and UK case studies

	Total interviewees / interviews	Gender		Age		Length of stay	
Japan	28 individuals	Men	17	20s	4	Range	11 m - 20y
	32 interviews	Women	11	30s	19	Mean	7.8y
				40s	5	Median	7y
UK	14 individuals	Men	4	20s	2	Range	10 m - 37y
	14 interviews	Women	10	30s	7	Mean	13.1y
				40s	3	Median	11.5y
				50s–60s	2		

The data for the Japanese part consist of formal interviews with 28 subjects from 12 CEE countries^3^ as well as data from participant observation at gatherings and informal meetings. The interviews were conducted as a part of larger project on Europeans living in Japan between 2011 and 2012, and in the second wave with follow-up and new interviews conducted between August 2019 and March 2020. The overall purpose of the project was to provide ethnographic data on first-generation Europeans living in Japan, focusing mainly on migration patterns, integration, and the role of whiteness in their integration. The interviews in both waves were semi-structured, covering mainly questions on migration to Japan, work and study experiences in Japan, social networks, and perceptions of integration in Japanese society.

The interviewees were recruited through a multi-nodal snowball sampling method resulting in a relatively diverse sample in terms of country of birth, length of stay, and occupation.^4^ The sample used in this paper aimed to account for migrants from CEE countries coming to Japan in different periods, using different visa schemes,^5^ and occupying different positions within Japanese society.

The data for the UK part consist of 14 semi-structured interviews conducted between 2018 and 2019 with 14 subjects from 5 CEE countries.^6^ As EU nationals, all participants had the right to free movement in the UK at the time of the interviews, including access to the labor market. Interviews are complemented by data drawn from participant observation at activist and cultural events focusing on CEE communities in Britain, including art exhibitions, performances, and events organized by migrant organizations. The data were gathered for a research project investigating CEE migrants’ narratives about race and migration in Britain, with a focus on historical memory. The interviews focused on experiences of being a migrant in the UK, the role of race and whiteness, CEE identity, and views on British people and other migrant communities.

The interviewees were recruited through social networks and events using snowball purposive sampling. They formed a diverse sample in terms of country of origin and length of stay in the UK and occupied a range of (largely middle-class) occupations.^7^ Due to the larger project aims, the sample is skewed toward interviewees with a predisposition toward reflection on the position of CEE migrants in the UK.

Self-identifying as white, coming from (EU-member) CEE countries, and having each lived in the respective country for approximately a decade allowed both researchers to share their own reflections on the topic of research with the participants and establish mutual rapport. Both studies were carried out in accordance with ethical qualitative research standards, ensuring that participants gave informed consent and remained pseudonymous, restricting access to their personal data, remaining aware of potentially sensitive topics, and offering interviewees the right to withdraw. Data analysis was conducted in two stages. In the first stage, interview data from each study were individually analyzed and coded. In the second stage, a new coding structure was created to ensure that the data used for the article addressed common questions. Selected anonymized excerpts of interview data were then shared according to the coding structure.

## Migrant visibility and differential constructions of white Europeanness

When data were compared, both studies emphasized the significance of CEE migrants’ visibility as one aspect of their contextual racialization. In the UK case, the position of CEE migrants was frequently linked by interviewees to the culturalist and geopolitical differentiation between Western and Eastern Europe. This view is exemplified by Katarzyna (Poland), who was scathing about her city council’s apparent lack of interest in tackling socio-economic inequalities faced by CEE communities in her local area. She was unconvinced by suggestions that the council preferred to classify all EU citizens in the same category due to their legal status, arguing that “everybody knows there’s a difference between East and West”.

Another interviewee, Iulia, who works for a migrant community organization, commented on the ambiguous role of whiteness in this differentiation. While for Iulia “racism” was an explicit aspect of the visibility of CEE migrants, she found its exact nature in relation to whiteness difficult to define:

“I think [that] Eastern Europeans in this country are kind of the other... I mean, not exactly white considered, you know? … They are, yeah, just some sort of like, white racism, or how is it called?”

Iulia, woman, 30s, Romania

On the one hand, the quote by Iulia implies that whiteness is not usually a target of racist hostilities in the UK, leading her to posit that “Eastern Europeans” are not “exactly” considered white. However, since this group is still subject to “white racism”,^8^ Eastern Europeanness is portrayed as an identity that subverts established color-coded rules about racism.

In addition to the East/West divide, the visibility of CEE migrants as a distinct group has to a large extent been shaped through discourses relating to social status (Böröcz & Sarkar, 2017; Paraschivescu, 2020). Although the nature of the UK sample means that most interviewees pursued typically middle-class occupations, primarily in education, the cultural industries, and the non-profit sector, they were hyper aware of the widespread perception of CEE migrants as low-skilled workers regardless of their own socio-economic position. One interviewee particularly emphasized the link between class, style, and visibility. He noted that while many people from CEE countries in the UK were “well-off” and “hipster-y”, there was another type of “Eastern European” who could be more easily identified in public spaces. In Nikola’s quote, a “tracksuit” is a symbol not only of a lower-class position, but also of more recent and less 'desirable' CEE migrants:

“‘That looks like an Eastern European with his tracksuit’ … And if somebody sees [my dad], they [would be like], ‘Oh my God, who’s this coming here taking our jobs,’ sort of type of thing.”

Nikola, man, 20s, Bulgaria

Nikola also drew on the first meeting between himself and the researcher in a Bulgarian community café as an additional example of CEE visibility that is not defined by phenotypic whiteness: “you were the most standing out character there, because you, I mean, you are an Eastern European, you’re white … but sort of even the way you dress or the way you look or the way your hair is, the way you sound … I saw you as a more Western Eastern European than all of the other people in that room”. The quote highlights that in the UK context, “Eastern Europeanness” has come to denote a particular form of embodied difference linked to social status, which includes but is not circumscribed by skin color.

In contrast with the UK, CEE interviewees in Japan do not see themselves as perceived differently from other white Europeans or Westerners, and are racialized largely as white based on phenotypic visible differences. The CEEs in Japan ‘discover’ their whiteness through migration, as they feel omnipresent differentiation in their everyday lives on the basis of skin pigmentation and other physical traits. During an interview, Jozef (man, 40s, Slovakia) remembered an almost 25-year-old utterance about his “white skin”, illustrating how an identity that many interviewees were previously unaware of comes to play a crucial role in their self-identifications in Japan. Jozef’s case also illustrates that while such differentiation does not erode with time or cultural and social integration, it does not necessarily have to be understood as a negative or even stigmatized identity:

“[I feel like] a foreign element [here in Japan] and I like it a lot. … I like being, not being part of that [Japanese] society, only to observe it from the outside.”

Whiteness bestows its bearers with positive connotations, and indeed, rather than seeing themselves as ‘migrants’, interviewees in their narratives referred to themselves most often as ‘foreigners’ or *gaikokujin* (or *gaijin*). This reflects the local discourse on migration and representations of ‘Others’ where the term ‘foreigner’ represents a more neutral or even positive term for a(n) (im)migrant. In contrast with the negative connotations of the latter (Roberts, 2018), the word *gaikokujin* is often (yet not exclusively) associated with whiteness as a symbol “largely desirable and worthy of envious emulation” in the realm of cultural representations (Russell, 2017, p. 27). It is also largely associated with an ideal type of desirable, high skilled and cosmopolitan migrant (Debnár, 2016, 2020; Hof, 2020, p. 14; Russell, 2017).

The visibility of Central-East Europeans as white ‘foreigners’ in everyday encounters mutes other forms of subtle differentiation of CEE migrants often seen in the UK and Western European context, such as those based on speech or accent, names, clothes, or nationality and ethnicity. Central-East Europeans in Japan are racialized not as “ambiguously white” (Lapiņa & Vertelyte, 2020) but simply as (white) Europeans where further distinctions are considerably limited. Similarly to Bojan (man, 30s, North Macedonia), many interviewees claimed in various ways that they “don’t think that the Japanese make the difference [between Western and Eastern Europe]”, and thus that the geopolitical and culturalist differentiation between Western and Eastern Europe have become largely irrelevant. In the following quote, Anton from Russia (man, 30s) claims that even in the case of Russia – which has a relatively distinct place in the local representations of the ‘Other’ in Japan as compared to other CEE countries – the (white) foreigner category (or *gaijin*) overarches the Russianness:

Feature of Russian people here in Japan, is that they are not really tight, [don’t] stay together. We can easily blend in *gaijin* community … Russian they don't stay together like people from Uzbekistan or Kazakhstan. Those kind of tribal kind of [people].

Anton suggests that there is a more cosmopolitan whiteness which is supposedly shared by Westerners, yet not typical for Asians, even those with shared Soviet history.^9^ While the category of ‘foreigner’ is often used inconsistently in general discourse, as it was by the interviewees, the cases above demonstrate how interviewees implicitly specify its tacit connotations and how the *gaikokujin* is often synonymous with whiteness, as well as the role played by tropes about racial Others (“tribal kind of people”) in maintaining racialized distinctions (Drnovšek Zorko, 2019).

Both case studies reinforce whiteness as a permeable and complex combination of visible differences and symbolic associations. In contrast with the UK, where internal divisions of Europeanness and stereotypes about unskilled labor play a significant role in determining CEE migrants’ visibility, in Japan, CEE migrants are included within the implicitly white and Western category of *gaikokujin*, a form of cosmopolitan whiteness that does not distinguish between East and West. Phenotypic characteristics such as skin color play a significant role in the visibility of ‘foreigners’ and point to the prevalence of biological racial logic in Japanese constructions of whiteness. Yet this racialization is accompanied by culturalist assumptions about behaviors such as not “staying together” in a tightknit community, or particular skill sets or language competencies presumably possessed by white Europeans (Debnár, 2016; Hof, 2020), which are reminiscent of the distinction linked to social status that are found in the UK. In the UK, although CEE migrants are deemed capable of approaching Western Europeanness through appropriate dress and mannerisms, being white is not sufficient to overcome the East/West divide, and the extent to which whiteness extends to ‘Eastern Europeans’ is questioned by some interviewees. In both cases, although CEE migrants’ whiteness has a different relationship to notions of Europeanness and its hierarchies, migration is key both to creating and maintaining racialized visibility.

## Racialized exclusions and negative representations

While ‘visibility’ is one aspect of the way that CEE migrants “emerge as uniquely racialized subjects” (Erel et al., 2016, p. 1347) in the two national contexts – as ambiguously white ‘Eastern Europeans’ in the UK and as implicitly white ‘foreigners’ in Japan – this does not capture the extent to which such racialization is experienced in relation both to the nation and to other migrant groups.

As already suggested, the whiteness of CEE migrants in Japan is constructed in the local context as a privileging identity that to a significant extent exempts its bearers from the negative connotations of Otherness and stigmatization associated with (im)migrants. Becoming invisible in their visibility empowers those perceived as white (Dyer, 1997) vis-à-vis other migrants, and many interviews more or less openly admit to their privileged position in a similar way to Jana (woman, 30s, Poland): “being a white [foreigner] is different, I think, different than [being an] Asian [foreigner]”. There was a shared perception that one of the main differences between the two is in the form of discrimination that they face as white migrants in Japan. Some would hesitate to even call the experiences of differentiation discrimination, and try to explain it rather as a “positive discrimination” (Anton, Ieva) or even “*gaijin* power”, as did Anastasia (woman, 30s, Ukraine), and Alena (woman, 40s, Slovakia) in the following quote:^10^

“Gaijin” is my hidden, very useful “weapon”, which I won’t let anyone to ever take from me.

Nevertheless, despite such recognition of white privilege by some of the interviewees, interviewees also shared the perception that the extent to which they can utilize their white privilege is limited, and that privilege does not imply inclusion (Debnár, 2016; Miladinović, 2020). Hof (2020) has argued that the whiteness of European migrants in Japan is “passive” – or “a symbol of status yet void of meaning” (p.16) – rather than a readily transferable form of social or economic capital. This elucidates further how whiteness is constructed in Japan as a privileging, yet at the same time alienating and exclusionary identity.

Depending largely on class, gender or life stage, the extent to which the interviewees interpreted their difference as negatively affecting their lives varied. On the other hand, the understanding that their difference is irrevocable and more or less alienating could be seen across the cases. The following quote demonstrates the ambivalence of being a visible ‘foreigner’ in Japan in a rather neutral yet still palpable way:

I don’t feel not accepted but I mean you do feel that you are a foreigner. That you look different and you are different. … So sometimes it's really like people [see you] positively, take you really positively but then again, it's again because you are a foreigner.

Szymona (women, 30s, Poland)

By pointing out that “you are [actually] different”, Szymona implies that being a white foreigner is not merely an issue of visibility but entails a racialized perception of ‘Otherness’. To be perceived as a foreigner in Japan remains something that define one’s whole life (Liu-Farrer, 2020, p. 149). Apparently privileging whiteness is confronted with the understanding of Japanese national identity with its “racial tenets” as an ethno-racially homogenous and culturally distinct entity (Kowner & Befu, 2015). The dissonance between the expectations the interviewees have regarding their social position, which are based on the notion of a hegemonic and privileging whiteness that CEE migrants ‘passively’ obtain by virtue of how their visible difference is interpreted in Japan, and the experience of perpetual ‘foreignness’ as an inherently alienating identity, is reflected in fumbling for the right words when trying to describe the place they believe they have in Japan. Variations on the often-heard elaborations as provided by Szymona above can be aptly summarized by largely positive yet quite vague expressions, such as a feeling that one is a “foreign element” (Jozef, Slovakia), “observer”, “always a tourist” (Marko, Croatia), “[not as] totally integrated, perfectly fitting in” (Bojan, North Macedonia), or just “that’s [actually] hard to say” (Jan, Czech).

Thus, on the one hand, Central-East Europeans in Japan become identified as largely unquestioned representatives of hegemonic global whiteness, which can be juxtaposed with the experience of CEE migrants in the UK, where such a claim to hegemonic whiteness is contested. On the other hand, CEE migrants in Japan are irreversibly excluded from belonging to the nation/majority. The awkwardness or apparent incomprehensibility lies in the fact that they are not excluded because their whiteness is being questioned, but precisely due to the acknowledgement of their whiteness without reservations and the realization that its ostensible hegemony and superiority are contested in the local setting.

In a parallel to the Japan case, interviewees in the UK study also reported feeling excluded, albeit in ways that aligned with the specificities of CEE representation in the UK context. Here Brexit and rising hostility against migrants represented a milestone for many interviewees (see also Botterill & Burrell, 2019; Rzepnikowska, 2019), with Iulia commenting that the differences between Central-East Europeans and Western Europeans “came out very clearly with Brexit and the referendum.” Furthermore, the awareness that CEE migrants are frequently portrayed in the media as “stealing jobs” or “benefit scroungers” led to a niggling sense of unease among interviewees that they were being treated differently in their own workplaces:

“Especially on the news, say, when they say anything about migrants or anything about Brexit it’s always, Polish people are always mentioned, ‘They come here in thousands and they’re taking your jobs’ … So, I don’t sometimes feel equal with the English teachers, I feel sometimes that they don’t treat me the same, maybe.”

Beata, woman, 40s, Polish

Reflecting on their place in British society, some interviewees were aware of their relative position vis-à-vis other explicitly racialized groups, both globally and within the UK:

“We, in the world divided by race, it feels like we have nothing to complain about, because we’re white. But there is a prejudice and there is a difference and we have our own culture which, surprise, is distinct from English.”

Ewa, woman, 30s, Poland

Although Ewa’s comment situates her and other CEE migrants within a globally privileged category of whiteness, she expresses ambivalence about white privilege as an entirely universal experience. In her quote it is a form of cultural racism that excludes CEE migrants from unproblematic acceptance into implicitly white Englishness, which has been identified as a central tenet of nostalgically imperialist visions of British sovereignty (Virdee & McGeever, 2018). Rejecting any assumptions about “putatively shared whiteness” (Fox et al., 2012, p. 681), Ewa expresses her non-belonging by sarcastically noting (“surprise”) CEE migrants’ difference from the English, and by extension from English whiteness.

Ewa’s remark highlights not only the prevalence of negative representation of A8 and A2 migrants, but also the lack of positive representation or knowledge about the region that could potentially incorporate these groups into a more pluralistic vision of Britishness, alongside other minorities and long-settled communities. As previous research has noted, the relationship between CEE and other migrant communities is not always one of solidarity or mutual acceptance (McDowell, 2009; Parutis, 2011). Iulia, when commenting on her professional work with CEE migrant communities, reflected that “the Central and Eastern Europeans, from when I go and talk to them, especially with the Romanian community, I’m afraid to say that people are quite racist towards other nationalities and other people from other parts of the world”. Yet she also highlighted instances where “simple folk, you know … coming from the countryside” arrive in the UK and “love it, they love the diversity”. Iulia further emphasized what she saw as divide-and-rule strategies on the part of the British that pit ‘old’ and ‘new’ migrants against each other:

“It’s coming from both ways, to be honest. I mean, the Indian and Pakistani community especially was very against Eastern Europeans. ... Turning us against each other, and that. … So it’s being fed, this whole ... “Oh, we hate these people,” “Well, we hate you as well.”

While interviewees in the UK struggle with what they see as a lack of positive representation in British society, their counterparts in Japan occupy a comparably more favorable position, not only in comparison with the UK but also in relation to Asian migrants. In both cases, however, ‘foreigners’ and ‘Eastern Europeans’ face struggles to inclusion. CEE migrants in the UK case – who represent a much larger share of the total population than in Japan – emphasize the changing political context of their exclusion. In contrast, in Japan the emphasis is on unassailable and enduring difference from the ethno-nationalist Japanese nation, which can be experienced as a form of alienation. When considering their place in each respective society, both groups also contend with existing assumptions about the global privilege of white Europeanness, especially in relation to other migrant groups.

## Conclusions

This article has analyzed constructions of whiteness in relation to CEE migrants in Japan and the UK, focusing on racialized visibility and exclusion. While ideas about race are always a co-production (Nowicka, 2018) of local and global dynamics, British and Japanese constructions of whiteness are rooted in the two countries’ different histories, patterns of immigration, and discourses of national belonging. By comparing a Western with a non-Western setting, we identify the specificity of receiving societies in shaping how whiteness is configured in changing contexts of migration, while not discounting the continued saliency of racialized meanings of ‘Europeanness’ and ‘Westernness’.

Our analysis highlights the importance of comparing such diverse contexts to understand how racialization as a process of differentiation is co-constituted through practices and discourses relating to migration, which has been termed the ‘race-migration nexus’ (Erel et al., 2016). While the comparison has its limitations and reveals important differences between the experiences of CEE migrants in the UK and Japan, it also shows commonalities in how racialization functions in relation to the visibility and exclusion of migrants. Visibility in both contexts is not limited to the biological logic of race, but includes culturalist assumptions and expectations about behavior or dress, although the former is more emphasized in Japan. Our analysis shows that while exclusion from the national norm or the assumed privileges of global whiteness is experienced differently and to varying degrees, it is significant to both groups’ experiences as ‘foreigners’ or ‘migrants’. In the UK, CEE migrants’ purported whiteness arguably eased their entry into the labor market but has been challenged by their stigmatization in the public sphere. In contrast, in Japan CEE migrants’ whiteness is not in question, but is precisely the ground on which their full inclusion in Japanese society is precluded.

Our findings further demonstrate the need for further attention to the social construction of race and whiteness in non-Western contexts, where racialized East/West dynamics are configured differently than in research on Europe or North America. In the UK, long-established internal European hierarchies as well as the changing context of migration politics have produced a racialized category of ‘Eastern Europeans’, a category with an ambivalent relationship to whiteness. In Japan, however, where intra-European distinctions are deemed less relevant, CEE migrants are perceived as implicitly white *gaikokujin* or ‘foreigners’, a category synonymous with Westernness. While the concept of race in Japan developed in relation to the West, which was viewed as desirable, discourses of Japaneseness also emphasize its difference and contest the idea of the presumed superiority of whiteness. The article therefore demonstrates the importance of comparing contexts such as Japan, where global whiteness as a privileging identity is reflected in local beauty or cultural standards (Ashikari, 2005; Russell, 2017) but where it is at least partially contested by local racial logics and hierarchies, with contexts such as the UK, where local meanings of whiteness are more directly aligned with global and regional hierarchies.

Finally, we call for further attention to how racialized visibility and exclusion are conceptualized in moments of crisis in both Japan and the UK. In 2020, Japanese initial measures related to containing COVID-19 resulted in banning (re-)entry to non-citizens, including permanent residents and foreign spouses or family members of Japanese citizens. This highlights the continued saliency of the ‘us and them’ distinction in Japan regardless of recent developments in immigration. We can compare this moment to the Brexit referendum, which revealed to many that the fault lines between ‘native’ and ‘migrant’ are based not only on legal status but are also varyingly racialized. Negative attitudes toward CEE migrants can further be juxtaposed with the necessity of importing seasonal migrant workers to pick fruit and vegetables during the strictest UK COVID-19 lockdown, and debates about who counts as ‘essential’ to the well-being of the nation (O’Carroll, 2020). Paying attention to such heightened moments of boundary-making can deepen our understanding of how notions of foreignness and belonging are conceptualized, as well as how they become attached to specific bodies.

## Data Availability

The datasets generated and/or analyzed during the current study are not publicly available due to personal data protection requirements.

## Footnotes

1 ‘Central-East Europe’ is not a precisely defined term and can include countries in Central, South-Eastern, and Northern (Baltic) Europe, and sometimes Russia. It is a geopolitical as much as a geographical concept used to denote countries that have historically been juxtaposed with Western Europe, particularly those that in the Cold War division of the world belonged to the ‘Second’ rather than the ‘First’ World. We use the term to include Russia as well as other ‘postsocialist’ countries both within and outside the EU. While we stress the heterogeneity of this region and the significant differences between individual countries, the article is interested precisely in the saliency or lack thereof of such geopolitical distinctions for the experiences of migrants.

2 While there are differences in, for example, exemptions from tourist visas or availability of working holiday visas between CEE countries, these differences are limited and not strictly reflecting EU membership. For instance, while EU nationals are exempted from tourist visas, they are required for some of the non-EU CEE nationals (CIS, Russia) but not from others (e.g., Serbia).

3 Interviewees were from eight EU and four non-EU (Russia, Ukraine, North Macedonia, Serbia) CEE countries.

4 Occupations included low-skilled factory worker, PhD student, English and other language teacher, engineer, manager, and university faculty.

5 All interviewees were in Japan with valid residency status.

6 Interviewees were from four EU (Romania, Bulgaria, Poland, Slovakia) and one non-EU CEE country (former Yugoslavia). The latter interviewee was in possession of British citizenship and was therefore not subject to immigration restrictions.

7 Occupations included shop assistant, university lecturer, nursery teacher, office administrator, and community worker.

8 In the context of the interview, the term “white racism” denoted “racism against whites” rather than the more common meaning of “racism perpetrated by whites”.

9 Such cosmopolitan self-identification can be understood as depending on Anton’s occupation (head-hunting for global specialists in Tokyo) as well as his gender. Some research on Russian women in Japan has suggested that different intersections of gender and class can lead to the more ‘ethnic’ practices referenced by Anton (see for example Kim, 2021).

10 The following quote from Alena, who was one of the informants for this project, is from a Facebook group post.
